# China and the global search for health security: history, vaccines, and governance

**DOI:** 10.1007/s42533-021-00066-y

**Published:** 2021-03-24

**Authors:** Daojiong Zha

**Affiliations:** School of International Studies, Institute of South South Cooperation and Development, Peking University, No. 5, Yiheyuan Road, Haidian District, Beijing, 100871 People’s Republic of China

**Keywords:** COVID-19, China’s history of influenza pandemics, Public health, Access to vaccines, International relations

## Abstract

China is a key player, not just an actor, in the global search for health security. Reiteration of this point is useful for International Relations studies, which often portray China as a factor to contend with, especially given the background of the country as the first to report the outbreak of the COVID-19 pandemic. This paper adopts an analytical framework developed through a summary of routines in Chinese engagement in global health from a practitioner’s perspective: aid, interdependence, governance and knowledge. These are the core elements in a country’s pursuit of engagement with the rest of the world. After the introduction, the second section of the paper reviews contributions from China in the history of global plague control over the past century. The third section discusses structural issues affecting access to vaccines, which are essential for bringing COVID-19 under effective control. The fourth section identifies a number of challenges China is facing in global health governance. The final section offers a few concluding thoughts, reiterating the nature of interdependence in the global search for enhancement of health security.

## Introduction

The purpose of this article is to offer a partial list of issues to consider in the renewed focus on China’s role in global public health during the unprecedented COVID-19 pandemic. In the immediate wake of China reporting the outbreak of a novel coronavirus, studies of the geopolitics of disease began to frame China’s nascent effort at disease control as “putting an alternative governance model to test” (Spinney 2020). It is true that epidemics and pandemics have always been political and involved the policing of borders, but the far-reaching effects of COVID-19 and the necessity for international cooperation and coordination demand more nuanced understandings of the roles that China and other states do and can play.

Health security encompasses human populations, animals, and the natural environment. With 19% of the total global population, and complex and large geographies of human and animal diseases within the country, China traditionally is a major referent of health security to the world’s professions of medical sciences, therapeutics and treatment. Linkages between China and the rest of the world, through trade and travel, mean that health events in the country can affect distant populations; conversely, health challenges originating in other countries can be imported into China with wide-scale effects. Indeed, China is a major corridor in the world’s infectious disease realm, in addition to being a major country in the domain of noncommunicable diseases.

In December 2019, China became the first country to report a new pneumonic syndrome that has since been named as a coronavirus disease (COVID-19) or a severe acute respiratory syndrome coronavirus (SARS-CoV-2), similar to but more severe than SARS of 2002–2003 (Coronaviridae Study Group 2020). By February 1, 2021, there were 102,083,344 confirmed cases of COVID-19 globally, including 2,209,195 deaths, reported to the World Health Organization (WHO) (https://covid19.who.int/). The pandemic is an unprecedented global public health disaster of a scale not seen in a century.

The international studies profession is producing a copious amount of literature contemplating COVID-19 and major powers’ maintenance and pursuit of security, influence, and status, against the backdrop of an increasingly complex China factor in global political-economic dynamics. Numerous think tanks are launching projects to contemplate the implications of different countries’ pandemic control and economic recovery efforts on changes in the balance of power and regional and world orders (Brands and Gavin 2020). Along such lines of thinking, the pandemic is a good crisis not to be wasted in the larger context of international politics, with power competition in the conventional sense of the term being the usual end product of assessment and analysis.

My treatment of this topic steers away from such conventional approaches in international studies. Instead, this paper contributes to understanding and analyzing the large and complex topic of China and international health security by drawing attention to a few structural issues central to considerations of international interactions in public health provision in general and pandemic response in particular. In the political history of plague control around the world, the outbreak of severe acute respiratory syndrome (SARS) in urban areas of China from 2002 to 2003 is viewed as the “first post-Westphalian pathogen,” implying that conventional reliance on and emphasis of a sovereign state as the primary actor is no longer adequate (Fidler 2003). Indeed, as a typical Western perspective observes (Hooker 2007, 179):

The SARS epidemic had the feeling of a watershed, a marker of historical change: on one hand, a return to an unhappy past when infectious disease threatened *Western nations* that had felt *free of epidemics*, and on the other, an optimistic transition to a global public health with the potential for swiftly identifying and containing in a place of origin any disease that might prove a threat to all (emphasis added).

COVID-19 certainly fits into the same line of mainstream international studies narratives about pandemics. Nonetheless, although both SARS and COVID-19 are pointedly associated with China as a country locality, the rationale behind the WHO’s guideline not to include “geographic locations: cities, countries, regions, continents” in naming a disease (WHO 2015) serves as a reminder that expertise in virology, rather than conventional considerations of the role of the state in international affairs, is what truly matters.

More to the point, with COVID-19 as a reminder to effectively address the exigencies of health in world affairs, international cooperation and coordination are a necessity rather than a choice. Even for a country that is less affected by the migration of a pathogen, such cooperation is conducive to improving its own preparedness for future public health disruptions. Quality of coordination among government agencies and international health institutions over the management of international travel, trade-in medical equipment, medicines, and the invention and deployment of vaccines greatly affects the pace of recovery to normalcy in international exchanges of human and material inputs for development. Diplomatic policies affecting cross-national epistemic communities’ capacity to exchange knowledge of therapies and health governance in a broader sense are just as significant. Actions in such areas, especially the provision of medical aid, can be viewed as relevant to a nation-state’s international prestige. However, health aid should be treated as an extended area of concern in international studies.

This paper adopts an analytical framework developed through a summary of routines in Chinese engagement in global health from a practitioner’s perspective. The framework lays out four pillars: aid, interdependence, governance, and knowledge. Health aid, traditionally flowing from developed societies to developing ones—but increasingly among developing economies as well (da Silva 2014)—contributes to global health equity. The notion of interdependence defines the pursuit of health security between countries as mutual protection against shared and transferred risks. Health governance, mediated by health diplomacy, is needed for setting ground rules of engagement. Knowledge as a pillar in managing global health includes the sharing of lessons in epidemic control and disease mitigation, in addition to its generalization and application worldwide as seen fitting by health professionals. Indeed, “knowledge centrally affects all four pillars of global health, and global health governance is recognized to be central to all four domains” (Liu et al. 2014, 794).

The second section of the paper offers a sketch of the knowledge pillar undergirding a country’s readiness for diplomatic/political cooperation concerning international health security. It invites attention to notable episodes in China’s struggles with major pandemic outbreaks over the past century. This is purposefully done because mainstream English language international studies literature tends to omit Chinese experiences as a source of contribution to the world’s search for effective pandemic response. That omission inadvertently affirms instincts of “us vs. them” in thinking about managing international cooperation to tackle COVID-19. Ahistorical treatment of the subject matter is unjustifiable because governments rely on public health expertise accumulated through experience from the past, including that gained from international exchanges.

The third section zeroes in on access to vaccines as a structural challenge in international health cooperation, partly because the deployment of safe and effective vaccines offers the best hope for ending restrictions on social interactions and international travel resulting from the spread of COVID-19. More significantly, the successful distribution of COVID-19 vaccines can become a modus operandi for managing international health cooperation in the future.

The fourth section outlines a few issue areas the author sees as meriting further research when thinking about China and international health governance, including in the wake of the COVID-19 pandemic. The paper eschews treatment of health aid—China as a recipient or a provider—as a separate issue. This can be justified because the topic has already received extensive treatment in the literature (Husain and Bloom 2020). The last section offers a summary of findings.

## Pieces of history: China in global efforts to control pandemics

History plays a constant and often contentious role in both vernacular and policy narratives about pandemics and responses to them. That contention begins with the association of a (new) pathogen with a place and/or country name. The world’s virologists are tasked with identifying a pathogen’s scientifically verifiable etiologic origin and epidemiological pathways of transmission. Customarily, such tasks do get entangled with consideration of nation-state interests, not least due to the associated societal stigmatization that occurs when connecting a pathogen with the name of a place or a country. In today’s networked world, that stigmatization, for any society, is considerable because of the possible negative implications such association can have for international travel and trade opportunities.

On February 8, 2020, China’s health authorities named the newly found virus in Wuhan, central China, as “novel coronavirus pneumonia.” The “COVID-19” naming by the WHO followed on February 11, and China immediately adopted it. Mainstream new media of Europe and North America were fast to drop prefixes such as “Wuhan,” “China,” “Chinese,” and/or “Asian” to the disease name (Prieto-Ramos et al. 2020). A small minority, including U.S. President Donald Trump, continued to use names criticized as discriminatory against China and/or Chinese/Asian cultures (Rogers et al. 2020). However, that was more a reflection of the diplomatic/political differences his government had with China over issues that are not directly related to control of COVID-19.

In the history of pandemic sociology, though, viruses were frequently associated with “China” or “Chinese.” For example, when investigating the geographical origin of the 1918 pandemic, some studies affirmatively speculated that Chinese laborers sent to France to participate in the First World War in Europe were the intermediate host (Langford 2005), while other studies continue to insist that such conclusions were tentative at best (Worobey et al. 2019). Two major influenza pandemics of 1957–58 and 1968–69, which spread across parts of Asia and Europe, continue to be called “Asian Flu” and “Hong Kong Flu,” respectively, in the media. Studies of historical pandemics are heavily affected by the availability of archival materials, which were severely limited to port cities accessible to European physicians and traders in the colonial era. This perhaps explains why, on the occasion of the centennial review of the 1918 pandemic, professional Chinese reviewers did not take issue with the naming of those flus of the past (Qin et al. 2018). Nor is the production of foreign and/or racial imaginary of a disease limited to the American or Western world. When the H1N1 influenza pandemic began spreading around the world in April 2009 and reached inside China a month later, public health professionals in Guangdong, the epicenter of SARS, reportedly concluded that H1N1 was “not a Chinese virus” (Mason 2015). Conceptual biases like this were also unhelpful for enhancing medical preparedness within China.

When it comes to COVID-19, the salient cost to China does not come from vernacular narratives of its origin, because the country’s priority is mounting an effective domestic response. Still, the prevailing imagery of China being nothing more than a source of disease and/or an epidemic-causing burden on the rest of the world is a cause for concern. As pathogens migrated around the globe long before the birth of the Westphalian system of world politics, which led to state-organized epidemic and pandemic response and health care systems, there is no scientific basis to set the record straight. The rest of this section, therefore, recounts recorded incidences in which China, as a locality, has indeed functioned as a source of solutions to common challenges in epidemic/pandemic response and, more broadly, disease control.

Study of a disease in the medical and life science profession involves the bacteriological, clinical, epidemiological, and ecological aspects of a pathogen. Identifying a new pathogen with a specific geographical location can also help to accumulate and spread scientific knowledge for its treatment and prevention. China has certainly benefited from the discovery of the plague bacillus by Alexandre Yersin in Hong Kong in the summer of 1894, although the spread of *Yersinia pestis* (named after the physician) included large areas of South Asia as well (Lynteris and Minor 2019). That discovery led to the understanding of bubonic plague as a virulent disease with a significant mortality rate, transmitted primarily by the bite of the rat flea (*Xenopsylla cheopsis*) or through person-to-person contact in its pneumonic form. The plague of 1894 is generally understood to be a key milestone ushering in the modern phase of disease control around the globe.

Indeed, the spread of knowledge about transmittable diseases from field epistemological work by European and Japanese physicians in southern China during the years before and after 1894 helped to coin the phrase *chuanran*—a Chinese term literally meaning disease spreads in human bodies as dye gets absorbed into the fiber of a cloth. That vivid reminder, in turn, became instrumental in promoting public awareness about animal-to-human disease transmission and self-protection through personal hygiene among the general populace. It was powerfully effective health education among a population with pervasively low literacy levels (Leung 2011).

Studies presented in the Chinese language about China’s long history of plague fighting are numerous (Deng 2006). While a language barrier is a reasonable cause for the paucity of English-language literature engaging Chinese experiences, it is nevertheless a pity that prevailing references to the 1918 influenza pandemic seldom mention China as a civilization/country that had its share of the same challenge and/or contribution. An extensive survey of academic databases held by the Peking University Library, one of the largest such holdings in China, yielded only two articles devoted to addressing the question of what happened in China during the 1918–20 global pandemic cycle. One study, based on primarily English language archives accessible to its authors, attributes the phenomenon to the possibility that the attack China suffered was relatively mild and less lethal than elsewhere in the world. It also speculates that the use of traditional Chinese medicine (herb-based), which understandably eluded recognition by Western physicians, was another cause for omission (Cheng and Leung 2007). In any case, the establishment of hospitals that practiced Western medical care in China began only after the Treaty of Wangxia (1844) was signed between the Qing Dynasty and the United States and was limited to port areas with concessions to foreign countries.

The other study, based primarily on Chinese language archives, finds the spread of the 1918 flu pandemic to be well beyond Hong Kong and ports in Guangdong province, as is sometimes mentioned in English language literature. Areas along the country’s coastline, including Shanghai and Zhejiang province, were also affected. The pandemic even reached as far as Yunnan province in the southwest and commercial centers in and near today’s Beijing. Archival accounts in the study reported higher death rates among youth populations and in the countryside compared to those in the more urban parts of the country (Alita et al. 2010).

Both accounts of the 1918 flu pandemic in China stress the tentative nature of their findings because the society of the day lacked a centralized governmental authority to systematically record public health events. The warlords in control of parts of northern China did set up a mechanism for plague control in 1919. However, the Nationalist Chinese government did not put a nominally national health bureaucracy in place until 1928, with coverage of a few large urban areas under its effective control. Vaccination as a means of epidemic control and disease prevention was introduced only in 1935 (Zheng et al. 2018).

In the modern history of fighting pneumonic plague, Chinese physicians’ success in controlling the Great Manchurian Plague epidemic of 1910–11 stands out as having enriched knowledge about disease control worldwide. In the winter of 1910–11, the Manchurian Plague outbreak had a 100% case fatality rate. In the scramble for treatment that involved medical professionals from China, Russia, Japan and France, Dr. Wu Lien-Teh (1879–1960) prevailed in the competition among physicians. He correctly ascertained the symptoms to be caused by a pneumonic plague by performing a postmortem on a victim who had died from contracting the virus. Wu, who had received training at Cambridge University, later went on to share his knowledge through writing in such medical journals as *The Lancet* and lecturing in Japan, the United States, and Europe, in addition to educating Chinese physicians about modern medical sciences (Goh et al. 1987).

In anthropological studies about the history of plague control, the Great Manchurian Plague is also known for the development and popularization of face masks as an anti-epidemic technology, which “Wu actively propagated as his own, personal invention” (Lynteris 2018, 444). Wu’s airborne contagion theory was contested by a French peer, who did not wear a face mask while operating in the adverse open-air conditions of wintertime Manchuria and subsequently died from contracting the virus. Pinpointing the precise origin of face masks as an anti-plague device, however, is not the key issue here. Instead, this episode clearly reveals that knowledge production for plague control did historically involve practices in China as well.

In terms of generating anti-epidemic knowledge, the 1910 bubonic plague in China’s war-torn Northeast is remembered for the first international conference in modern China to share and debate expertise in clinical as well as epidemiological aspects of animal-human transmission of viruses. The conference, held in April 1911 in Shenyang, marked the beginning of the debates about philosophical and clinical aspects of Chinese and Western sciences of medicine (Gamsa 2006). Incidentally, similar debates are continuing today over the overwhelming reliance on traditional Chinese medicine as a therapeutic solution in Fangcang shelter hospitals in Wuhan, the epicenter of COVID-19 in China (Chen et al. 2020).

What present-day relevance does such recounting of history have for discussions about pandemic control today? One possible point of generalization is that for China’s medical and public health professions, past experiences and lessons, including those gained through international exchange, are valuable referents for controlling the recurrence of infections. For example, in November 2019, Chinese doctors at a hospital in Beijing identified traces of infection suspected to be related to the same bacterium *Yersinia pestis* as the one that caused the plague a century back. Within days, effective treatment was rendered in the visiting patients’ hometown in Inner Mongolia, thousands of miles away (Xinhua 2019).

In viewing health as an international relations topic, one must bear in mind that the accumulation of knowledge about viruses and diseases and the pursuit of progress in medicinal and clinical sciences between China and the rest of the world have not and should not have modern demarcations of nation-state boundaries as barriers. Statements in international studies literature about COVID-19 that cast China as an outlier are devoid of historical facts concerning the evolution of health knowledge around the world. In short, discussions about China’s role today in the search for international health security can benefit from knowledge about the past. Disease control has always entailed international exchange and cooperation.

## Access to vaccines

Effective treatment of a pneumonic disease such as COVID-19 has to begin with ensuring that critical personal protective equipment (PPE) products are sourced and allocated to frontline health workers and other responders in affected areas. But the world’s PPE supply chain has not functioned properly to meet a surge in demand due to constraints in production and logistics (ADB 2020).

Over the years, as China has emerged as the world’s second-largest healthcare market after the United States, international pharmaceutical and medical device firms relocated production to the country to take advantage of lower labor costs in production and to more economically serve the needs of the local population. One account reports China to have “provided 43 percent of world imports of face shields, protective garments, mouth-nose-protection equipment, gloves, and goggles in 2018,” the year before the outbreak of COVID-19 (Bown 2020).

When COVID-19 was first detected in China, some of the world’s PPE inventory was sold and donated to meet the sudden surge in Chinese demand. Disruptions to cargo transportation resulting from government curtailment to interrupt the spread of the virus through human and cargo traffic also complicated the functioning of supply chains (Harvey 2020). The Chinese government responded by investing in massive production of PPE. As the supply shortage of PPE within China became less acute, China began to export it. Meanwhile, the deployment of PPE products in other countries needed to be approved through emergency use authorizations by their regulatory agencies, which must deal with a complex web of international, regional, and country standards (WHO 2020). China’s role as a supplier became controversial. Criticism surrounding China’s practice of “mask diplomacy,” is partly attributable to incompatibilities of technical standards. That criticism is also a reflection of “the dividing lines between advocates of engagement and containment with the country” (EIU 2020, 32).

But it is vaccines that are truly essential for a society to strike the balance between reducing disruptions to normal economic activities and daily life and protecting the health of the entire population. Although the severity of the pandemic differs from country to country, each society has to eventually move from containment to the treatment phases of COVID-19 control.

The onerous reality is that vaccine development is difficult, complex, highly risky, and costly. It includes clinical development, process development, and assay development. The risk is high because most vaccine candidates fail in preclinical or early clinical development and less than one in fifteen vaccine candidates entering Phase II achieves licensure. Full licensing requires demonstration of safety and effectiveness during Phase III clinical trials. After a vaccine is approved and released, optional Phase IV trials continue to test the vaccine for safety, efficacy, and other potential uses. Under normal circumstances, a successful vaccine takes up to fifteen years to complete a development cycle (Plotkin 2018).

To develop a COVID-19 vaccine, scientists around the world immediately began analyzing genetic sequencing data publicly posted by Yongzhen Zhang of the Shanghai Public Health Clinical Center & School of Public Health, Fudan University, on January 11, 2020 (Cohen 2020). According to a timeline of events published by the Xinhua News Agency, on January 24, 2020, the WHO acknowledged receipt of relevant genetic data China had shared (Xinhua 2020). More data became available as cases began to appear around the world, enabling laboratory research for designing vaccines. By the middle of 2020, governments in Europe and the United States started to place advance orders for hundreds of millions of doses of vaccines upon successful development. Although such monetary commitments can incentivize vaccine developers to take on risk, at least in the initial stage of successful development, vaccines will be limited in supply, given the global spread of the pandemic. Poorer parts of the world stand to suffer from practices of “vaccine nationalism” (FT 2020).

Concerns about lack of equal and equitable access to vaccines are valid, because even without the disruption of a pandemic, people in many countries, especially those in the developing world, traditionally struggle to gain timely access to vaccines for new diseases. As noted by the WHO (WHO undated):

About 80% of global vaccine sales come from five large multinational corporations…Many of the individual vaccine markets are monopolies or oligopolies, either by product or presentation. The limited number of vaccine suppliers and production capacities leads to a tenuous balance between demand and supply in many individual vaccine markets.

International organizations, since the mid-1970s, have played a key role in expanding vaccine access worldwide, with the WHO certifying prequalified products for countries without indigenous development and manufacturing capacities to procure from a pool of supply. The United Nations International Children's Emergency Fund (UNICEF), in addition to philanthropic entities, also assists by securing supply from multinational vaccine producers and offering the products to developing countries as donations and/or at discounted prices. In turn, many developing countries enter into advanced purchase guarantee arrangements for suppliers to deal with potential losses of income due to mutation of the targeted viruses or other medical circumstances that warrant termination of vaccine use.

The critical issue in the international circulation of vaccines is the physical availability of supply. With “government use” being a principle of action allowed under international medicine trade arrangements such as Trade-Related Aspects of Intellectual Properties (TRIPS), history is replete with examples of developed countries exercising sovereign exclusivity—including among friends and allies—over influenza vaccines that populations in many countries would have to rely on (Fidler 2010).

A prime example of “vaccine nationalism” in the recent past is competition for access to H1N1 vaccines in 2009. Developed countries placed large-scale, advanced marketing commitments for it and bought virtually all the vaccines that companies could manufacture. The WHO negotiated with vaccine manufacturers and developed countries to secure some vaccines for developing countries. The WHO and the United Nations (UN) also appealed for monetary donations to purchase vaccines and other supplies for use in affected low-income countries. Manufacturers did agree to make donation pledges, “but the donations still left the developing world with limited supplies, compared to developed countries, which would retain, even after donations, sufficient vaccine to cover their populations” (Fidler 2010). During the 2009 H1N1 influenza pandemic, the United States and many European countries donated 10% of their vaccine stocks to poorer countries, but only after it became clear they had enough for their own populations. During the H1N1 pandemic in 2009, developing countries lucked out due to the unexpectedly short duration of the disease’s spread.

To avoid a repetition of the 2009 H1N1 scenario, in early June 2020, the WHO, Global Alliance for Vaccines and Immunizations (GAVI), and Coalition for Epidemic Preparedness Innovations (CEPI) jointly set up a system to accelerate and equitably distribute vaccines, known as the COVID-19 Vaccines Global Access (COVAX) Facility. The goal is to have 2 billion doses by the end of 2021, enabled by advance purchase commitments and donations from governments and international organizations. Under the scheme, dosages are to be sold according to a country’s per capita income. High and upper-middle-income countries pay the full price which is agreed upon with manufacturers, whereas low and lower-middle-income countries are asked to purchase at subsidized prices. At the time of this writing, 190 countries have joined the COVAX Facility.

China, with its own vaccine candidates in the race, participated in demonstrations of solidarity such as the Global Vaccine Summit on June 4, 2020, at which world leaders including those from Canada, Germany and the United Kingdom, together with the Bill and Melinda Gates Foundation, pledged to make financial contributions and offer millions of doses of COVID-19 vaccines as part of the Gavi COVAX Advance Market Commitment (Lancet 2020). Then, in October 2020, China announced its decision to join the COVAX Facility.

China’s acts came on the heels of President Xi Jinping offering to provide $2 billion over two years to support the fight against the COVID-19 pandemic in a speech to the World Health Assembly (the governing body of the WHO) in early May 2020. Xi announced that China was going to make its COVID-19 vaccines, when successful, a public good for global health. He also stated that ensuring vaccine accessibility and affordability in developing countries was a goal of Chinese diplomacy.

It should be noted that China is still on the lower echelon of the international hierarchy of the vaccine industry, which has developers and manufacturers in GAVI at the top. Chinese practices of inoculation and vaccination have hundreds of years of recorded history. But it was not until 2013 that China had a Chinese-made vaccine (for a strain of encephalitis) prequalified by the WHO. It was nonetheless the official beginning of the Chinese vaccine industry entering the world market as a supplier and licenser (WHO 2014). This achievement was possible after decades of collaboration with PATH (Programs for Appropriate Technology in Health), a coalition of medical and business interests headquartered in Seattle, Washington. PATH in turn works in close collaboration with GAVI (Stevenson 2018).

China’s pledge to make its vaccines a public good for global health also results from necessity in vaccine development. The country’s swiftness in bringing the pandemic under control left it short of infected patients to carry out Phase III trials. As a result, Chinese candidate vaccines are being tested in over a dozen developing countries, including populous ones like Brazil and Indonesia. When Chinese COVID-19 vaccines successfully acquire prequalification status from the WHO, they will be eligible for inclusion in the COVAX Facility for distribution including among countries that have not participated in the test trial stage of development.

In addition, China can and indeed should play a more active role in the Developing Countries Vaccine Manufacturers Network (DCVMN). For example, through this platform, China could license the production of its vaccines in other developing countries and make available its own manufacturing capacities to mass-produce vaccines developed abroad under proper licensing. As a mechanism, the DCVMN can make up for relative weakness in development by facilitating massive levels of manufacturing to meet the presumed high demand. A speedy transition from a proven vaccine to large-scale manufacturing will be essential in order to commence immunization globally as soon as possible. The right mixture would also require time, prequalification by the WHO, and certification by national regulatory authorities. In any case, fuller utilization of the world’s vaccine manufacturing capacity is conducive to the goal of reducing per-unit costs and thus enhancing their accessibility for low and lower-income countries.

Hopefully, China’s proactiveness toward COVID-19 vaccines becomes a new normal in its approach to international health affairs. Overall, for the sake of managing COVID-19 and international health security in general, at the very least, when a country as populous as China moves more firmly in the direction of materially ameliorating a global health emergency, stress on the entire world can be reduced. In terms of global health governance, this is more affirmation that expertise-based international cooperation rather than competition for international political status is what truly matters.

## China in global health governance

COVID-19 is bound to usher in more efforts to examine structural issues in global health governance and China’s role therein. Scope for such inquiry is large, and analysis can benefit from health-related public policy studies in the multiple disciplines of social science. China’s position as a major corridor of both infectious and noncommunicable diseases makes the country a necessary consideration in the search for more encompassing global health governance mechanisms. This section introduces several issues that are foundational for international studies research to pay attention to.

The first issue is what lessons can be drawn from China’s evolving engagement with international health development institutions. On the one hand, the WHO, the Global Fund to Fight AIDS, Tuberculosis and Malaria, and the World Bank, in addition to bilateral aid from developed country governments and international philanthropic agencies, have played sustained roles in China’s health system development since the early 1980s (Szlezak 2012). On the other hand, there have yet to be systematic reviews of how China’s health agencies have engaged with the WHO, which established a representative office in Beijing in 1981.

It is known, for example, that China failed to utilize resources the WHO could have assembled to assist the country in combatting SARS, in addition to underutilizing the significant virology expertise already domestically available (Enserink 2003). International health practitioners’ recounting of efforts to tackle SARS seems to indicate that the WHO operated autonomously (MacKenzie et al. 2004). Collaboration with the WHO seems to have improved a decade later, with China effectively controlling the H7N9 influenza virus within its borders in 2013 (Wang 2013). Still, a relatively systematic study of China’s involvement in global health governance in the 2000s concludes that its interest tilted toward gaining access to the world’s medicine markets (Lee and Chan 2014). The author learned through an interview with a retired Chinese national with years of work experience in the WHO system that such inquiry will have to rely more on the recollections of knowledgeable individuals because the undigitized archives of the WHO’s Beijing office are quite possibly incomplete due to limited storage space (Interview 2020).

Still, it is puzzling why upon the outbreak of COVID-19 in Wuhan in the winter of 2019, the WHO seemed to have played an advisory role at best. Enlisting assistance from the WHO ought to have been a natural course of action especially after ten years of a Chinese national—a veteran public health official credited for effective management of SARS in Hong Kong—holding the director generalship of the WHO for two consecutive terms (from 2007 to 2017). Serious efforts need to be made to appraise the quality of China’s interactions with the WHO and other international health agencies, to promote effective communication and interaction to better address health emergencies and the everyday needs of a quarter of the world’s population.

The second issue that warrants more attention is how China itself relates to the host of bodies and entities that make up the international pharmaceutical industry sector. Measured by per capita income and official foreign policy position, China as a middle-income country is in a position to take advantage of health-related provisions under the TRIPS regime. But China neither makes use of the flexibility accorded by TRIPS to produce more affordable medicines for impoverished patients in the developing world nor takes steps to put forward systemic changes in the agreement in favor of developing countries. Instead, China opts to conduct tendering and negotiations with multinational drug companies seeking to enter its pharmaceutical product markets (Verghese et al. 2019). As a matter of fact, many other developing countries opt to utilize the threat of compulsory licensing as leverage to increase country access to essential medicines (Ooms and Hanefeld 2019).

Furthermore, China is one of the very few major economies in the world that does not make pharmaceuticals a separate component in its free trade agreement (FTA) negotiations. In recent years, voices on social media emerged in the country over unaffordable brand name anti-cancer drugs and the seemingly unconcerned response of the Chinese government to public discontent. The Chinese government’s seeming unwillingness to make use of the FTA mechanism or to invoke medical flexibility under TRIPS can be explained by its preference for the WTO as the dominant institution for global economic lawmaking and dispute resolution. It seems to be apprehensive of pushback from a broad range of industries should it be viewed as joining forces with other countries that have actively pursued compulsory licensing of patented medicines (Jiang 2019). How COVID-19 affects government thinking and generates potential policy change merits continued research attention.

The third issue arises from the fact that China continues to be relatively weak in the world’s epistemic communities in health, whose expert advice plays a major role in standard setting through professional associations (Lee and Chan 2014). The cause can be partially attributed to the short history of China’s engagement in global health, compared with that of other major countries. Its health aid is overwhelmingly concentrated in Africa, and in a few selected areas such as malaria control. Its knowledge exchange efforts with developed countries heavily feature those funded abroad for disease and medicinal data collection, in addition to demonstration projects to assist sales of medicines and equipment. Public health as a discipline of tertiary education in China has a short history and Chinese language proficiency poses a formidable barrier for deep communication among participating students and scholars. Last but not least, the Chinese pharmaceutical industry is still catching up in the international hierarchy, limiting the extent to which it influences the world’s industry shapers of global health governance policies (Liu et al. 2014).

Challenges in handling COVID-19 around the world ought to motivate more extensive and in-depth convergence between Chinese and foreign epistemic communities in health. Future efforts to increase Chinese involvement in international public health must rely on broadening and strengthening an epistemic community that forms the backbone of expertise-based country representation in international health governance. A good deal of effort in this regard will have to come from an enabling government policy environment for a professional association with minimal interference from diplomatic entanglements. Studies on China’s international relations can contribute through increased immersion in public health as a body of knowledge and by encouraging greater appreciation of human welfare (as opposed to conventional understandings of national security) as a driver of interactions between China and the rest of the world.

The fourth issue concerns the prospect of spillover effects on public health in China resulting from economic sanctions by sender countries such as the United States. As part of the deterioration of bilateral diplomatic and economic relations under the Trump administration, the dynamics of cooperation between the centers of disease control and prevention of the two countries, which strengthened in the wake of the SARS epidemic in parts of China in 2002–2003, the dynamics of exchange came to a halt. Under Trump’s “America First” doctrine, health cooperation with China was viewed as a loss because it supposedly assisted China to become a more capable rival to American power, influence, and status. This situation openly challenges the logic of international health as providing mutual protection and improving a country’s national security (Zha 2021).

Pharmaceutical products and medical equipment have thus far been spared from the United States’ sanctions against China. Nor have Chinese corporations of medicine and health care equipment been put on American “entity lists” and therefore become subject to sanctions. But China has good reasons to be worried about the future prospect of adversarial economic sanctions by the United States, especially since the punitive regime can easily gain a life of its own once set in motion.

Biotechnology is already a contentious area of competition between China and the United States and other industrialized economies. In “Made in China 2025,” an initiative that already caused international protest and opposition, biomedicine and bio-based materials are specifically considered parts of advanced manufacturing. With the United States working to constrain Chinese access to advanced semiconductors, including through secondary sanctions on suppliers of non-U.S. origin, adverse impact on Chinese progress in biomedicine manufacturing is an almost certain side effect.

Studies of public health consequences from economic sanctions, including those designed with humanitarian exemptions (usually food, medicine, and medical supplies), “economic coercion might still inadvertently harm the physical well-being of civilians” (Peksen 2011). Whereas China is far more developed and resourceful in withstanding the effects of economic sanctions compared to countries like Cuba, Haiti, Iran, and Iraq—all traditional targets of economic sanctions by the United States—it cannot expect to escape international restrictions on the purchase of medical equipment and pharmaceutical products and their damage to its health infrastructure.

A causal linkage between a country’s access to advanced technology abroad and change in its public health situation is difficult to establish short of medicine and/or medical equipment being specifically included in economic sanctions. However, a lesson that ought to be drawn from the global spread of COVID-19 is that health effects from economic sanctions should no longer be viewed as an unintended and unavoidable consequence. Scholars of international studies should draw insights from COVID-19 generated emergencies over PPE to assess international connectivity in standards and quality control and promote competition through innovation, regardless of the nationality of an invention or a health product.

Admittedly, this paper’s analysis is China-centric and avoidance of unintended negative consequences on public health will depend as much input from China as it does from other countries. It is therefore essential to consider these and other issues at the operational level of global health security, rather than abstractly discussing competition over vaguely defined national interests. When it comes to public health, identification of a country’s national interests must be based on expert input from health professionals, instead of grand theorization about future events given world trends.

## Conclusion

Without a doubt, COVID-19 qualifies as a harbinger of new approaches to global governance in search of enhanced health security for humans, animals, and the environment. As stated at the outset, this paper’s goal is to serve in a small way to encourage more thinking about China as a naturally inclusive participant in the world’s search for more effective means to address epidemics, pandemics, and other aspects of public health. Studies of public health and global governance differentiate between health as a venue for governance (with geopolitical connotations therein) and refinement of diplomatic and international economic governance for promotion of health (Ng 2014). This paper sees more value in the latter perspective.

The ongoing COVID-19 pandemic will eventually end, either by the expiration of the pathogen’s lethality, or by effective intervention by governments and societies around the world. In its wake, interactions between China and the rest of the world through travel and trade will resume. Hopefully, my sketch of China *in* global campaigns against regional and global pandemics can help to guard against the recurrence of “us-vs.-them” sentiments, however, subliminal, in international relations approaches. Competition is a rule of the game in interactions between nation-states and societies. But when it comes to public health, arguments in support of cooperation and coordination must prevail, simply because future pathogens will continue to disregard nation-state boundaries.

Due to the author’s knowledge and professional limitations, the effects of diplomatic and geopolitical contention concurrent to the unfolding of the COVID-19 pandemic between China and major countries on public health interactions is difficult to assess. In this paper, recounting of such interactions prior to interventions to limit intercontinental travel is partial and patchy. Still, it will be desirable for international studies to positively contribute to policies that enable resumption and expansion of such activities. After all, more convergence between countries in virology, medical sciences, and therapeutic practices and norms is conducive to both individually and collectively withstanding future shocks to global public health.

The world is unevenly but definitively moving from the response to the recovery phase in the management of the COVID-19 pandemic. The necessity and utility of international assistance, particularly in the form of donated medical supplies and equipment, has exhausted its due trajectory. Equal and equitable access to vaccines has become a clear and present challenge. A combination of unilateral, bilateral, and multilateral means is setting in, accompanied by calculations of possible gains and losses in a country’s soft power. International studies ought to encourage multilateral pooling of resources to facilitate health mitigation and preparedness (for the next pandemic and everyday health provision), taking its lack of such thus far over the COVID-19 shock as a lesson.

Last but not least, it would be fallacious to suggest that concern about global public health alone can become a pivotal factor in the conventional treatment of relations between states as being fundamentally about competition and trust. The world’s health professions evolve with their own cooperative and competitive elements therein, with competing interests in investment, research and development, marketing, and national prestige. To borrow a term from clinical science, underlying conditions in accumulated political-diplomatic impulses behind interactions between China and the rest of the world need to be identified and treated as well. Economic sanctions, including those on access to technologies, stem from those underlying conditions and can adversely affect the receiving society’s contributions to virus surveillance and analysis and epidemic preparedness. Containing fallout on public health provision in the receiving country should be on the agenda for international studies research.
